# Supplementary material for: Respiratory Adsorption of Organic Pollutants in Wastewater by Superhydrophobic Phenolic Xerogels
Source: Polymers (Basel). 2022 Apr 14;14(8):1596. doi: 10.3390/polym14081596 (PMC9028260; doi:10.3390/polym14081596)
Supplement: Supplementary file 1 [file polymers-14-01596-s001.zip › Supplementary Information.pdf]

Supporting Information

Respiratory adsorption of organic pollutants in wastewater by superhydrophobic phenolic xerogels

Yinchun Li [1,+], Depeng Gong [1,+], Youliang Zhou[1], Chaocan Zhang [1,*], Chunyang Zhang[1], Yitian Sheng[1], Shu Peng[1]

[1] School of materials Science and Engineering, Wuhan University of Technology, Wuhan 430070, China; liyinchun@whut.edu.cn (Y.L.); gdpyy1@163.com (D.G.); zyl_2013@whut.edu.cn (Y.Z.); polymers@whut.edu.cn (C.Z.); 13476089628@163.com (C.Z.); 969107575@qq.com (Y.S.); 404913513@qq.com (S.P.)

* Correspondence: polymers@whut.edu.cn (C.Z.)

+These authors contributed equally to this work.

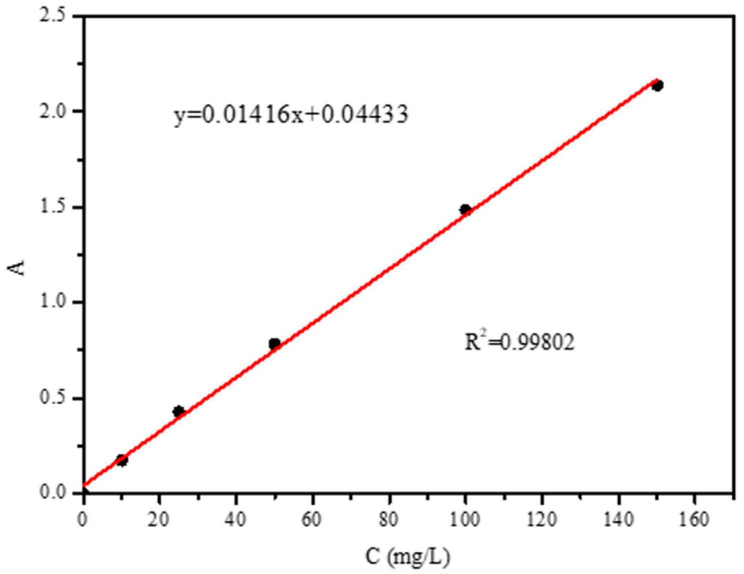

Figure S1. Standard curve of phenol aqueous solution.

Table S1. Properties of xerogels with different solvent systems

Solvent	Catalyst	Phenomenon and Conclusion
Ethyl acetate	HCl	Poor compatibility with resin, milky white solution, insufficient cross-linking, heavy granularity of gel, inelasticity
2-Methoxyethanol	HCl	Good compatibility with resin, light yellow transparent solution, white gel, slight granularity, large gel shrinkage
1,2-Dimethoxyethane	HCl	Good compatibility with resin, light yellow transparent solution, after adding catalyst for a period of time, only phase separation without gelation, forming a large molecular structure dissolved in the solution
Diethylene glycol	HCl	Good compatibility with resin, light yellow transparent solution, gel first and then phase separation, no obvious particles, no elasticity and brittleness
Ethylene glycol /1,2-Dimethoxyethane	HCl	First phase separation and then gelation, and with the increase of the amount of ethylene glycol dimethyl ether, the gelation time after phase separation will be prolonged. The more the proportion of ethylene glycol, the greater the elasticity, the more the proportion of ethylene glycol dimethyl ether, the more obvious the system shrinks, and the stronger the graininess.
Ethylene glycol / Diethylene glycol	HCl	The gel is first separated and then gelled. The higher the proportion of ethylene glycol, the softer and more elastic the gel. With the increase of the content of diethylene glycol, the elasticity of the gel becomes smaller and brittle.
Ethylene glycol	HCl	Good compatibility with resin, light yellow transparent solution, wet gel is white, no obvious graininess, first phase separation and then gel, with good elasticity
Ethylene glycol	P-toluenesulfonic acid	The organic acid system gel is relatively fine, the pore size is small, and there is no elasticity
Ethylene glycol	Organic and Inorganic Acid Compounding	The elasticity is smaller than that of the inorganic acid system and gradually decreases with the increase of the proportion of organic acid
